# Porphyrin Photosensitizers into Polysaccharide-Based Biopolymer Hydrogels for Topical Photodynamic Therapy: Physicochemical and Pharmacotechnical Assessments

**DOI:** 10.3390/gels10080499

**Published:** 2024-07-27

**Authors:** Andreea Mihaela Burloiu, Emma Adriana Ozon, Adina Magdalena Musuc, Mihai Anastasescu, Radu Petre Socoteanu, Irina Atkinson, Daniela C. Culita, Valentina Anuta, Ioana Andreea Popescu, Dumitru Lupuliasa, Dragoș Paul Mihai, Cerasela Elena Gîrd, Rica Boscencu

**Affiliations:** 1 Faculty of Pharmacy, “Carol Davila” University of Medicine and Pharmacy, 6 Traian Vuia St., 020956 Bucharest, Romania; andreea-mihaela.burloiu@drd.umfcd.ro (A.M.B.); valentina.anuta@umfcd.ro (V.A.); andreea-ioana.popescu@umfcd.ro (I.A.P.); dumitru.lupuliasa@umfcd.ro (D.L.); dragos_mihai@umfcd.ro (D.P.M.); cerasela.gird@umfcd.ro (C.E.G.); rica.boscencu@umfcd.ro (R.B.); 2 Institute of Physical Chemistry—Ilie Murgulescu, Romanian Academy, 060021 Bucharest, Romania; manastasescu@icf.ro (M.A.); iatkinson@icf.ro (I.A.); dculita@icf.ro (D.C.C.)

**Keywords:** unsymmetrical porphyrin, hydroxypropyl cellulose, porphyrin photosensitizers, hydrogel formulations, topical photodynamic therapy, physicochemical characterization, pharmacotechnical evaluation

## Abstract

Photodynamic therapy (PDT) is an emerging treatment modality that utilizes light-sensitive compounds, known as photosensitizers, to produce reactive oxygen species (ROS) that can selectively destroy malignant or diseased tissues upon light activation. This study investigates the incorporation of two porphyrin structures, 5-(4-hydroxy-3-methoxyphenyl)-10,15,20-tris-(4-acetoxy-3-methoxyphenyl) porphyrin (P2.2.) and 5,10,15,20-tetrakis-(4-acetoxy-3-methoxyphenyl) porphyrin (P2.1.), into hydroxypropyl cellulose (HPC) hydrogels for potential use in topical photodynamic therapy (PDT). The structural and compositional properties of the resulting hydrogels were characterized using advanced techniques such as Fourier-transform infrared (FTIR) spectroscopy, X-ray diffraction (XRD), thermogravimetric analysis (TGA), atomic force microscopy (AFM), UV-Visible (UV-Vis) spectroscopy, and fluorescence spectroscopy. FTIR spectra revealed a slight shift of the main characteristic absorption bands corresponding to the porphyrins and their interactions with the HPC matrix, indicating successful incorporation and potential hydrogen bonding. XRD patterns revealed the presence of crystalline domains within the HPC matrix, indicating partial crystallization of the porphyrins dispersed within the amorphous polymer structure. TGA results indicated enhanced thermal stability of the HPC–porphyrin gels compared to 10% HPC gel, with additional weight loss stages corresponding to the thermal degradation of the porphyrins. Rheological analysis showed that the gels exhibited pseudoplastic behavior and thixotropic properties, with minimal impact on the flow properties of HPC by P2.1., but notable changes in viscosity and shear stress with P2.2. incorporation, indicating structural modifications. AFM imaging revealed a homogeneous distribution of porphyrins, and UV-Vis and fluorescence spectroscopy confirmed the retention of their photophysical properties. Pharmacotechnical evaluations showed that the hydrogels possessed suitable mechanical properties, optimal pH, high swelling ratios, and excellent spreadability, making them ideal for topical application. These findings suggest that the porphyrin-incorporated HPC hydrogels have significant potential as effective therapeutic agents for topical applications.

## 1. Introduction

Topical photodynamic therapy (PDT) is a non-invasive technique used successfully in the treatment of both malignant and non-malignant skin diseases [1,2]. PDT is a highly targeted therapeutic method that provides the benefit of field PDT, thus reducing the potential for systemic phototoxicity [1,2,3]. The method involves applying a photosensitizing agent directly to the affected area of the skin, followed by irradiation with red light, and the generation of a localized cytotoxic species in the presence of molecular oxygen resulting in cell death and tissue destruction [1,4,5].

Porphyrin, chlorine, and bacteriochlorin derivatives are widely recognized and commonly utilized photosensitizers (PS) in photodynamic therapy of cutaneous diseases [3,6]. Thus, a series of pharmaceutical products containing tetrapyrrole-based photosensitizers (Metvix^®^, Levulan^®^, Photogem^®^, Purlytin^®^, Foscan^®^, Foslip^®^) are currently used in topical photodynamic therapy. Tetrapyrrole structures have good selectivity for tumor cells, low cytotoxicity in the dark, excellent photophysical properties with a high molar absorption coefficient in the phototherapeutic window (600–800 nm), and good efficiency in promoting reactive oxygen species [7,8,9].

Despite these advantages, the application of porphyrin derivatives in topical PDT has been limited because of their reduced solubility in biological media and molecular aggregation tendency with a negative effect on uptake in tumor cells [10,11]. Due to the poor penetration of photosensitizer into the cutaneous tissues, the therapeutic effectiveness of topical PDT will be limited only to superficial skin lesions. To overcome these difficulties, several strategies have been approached in the last few years. 

The structural modifications in PS architecture can suppress the molecular aggregation tendency and can improve PS penetration into deep epidermal regions with a good cellular internalization. The structural optimization of the photosensitizer molecule is carried out in a chemo- and regioselective manner by the attachment at the tetrapyrrole ring of functional groups with various polarity degrees, which can increase its solubility in the biological environment, decrease aggregation tendency, and improve photodynamic properties for PDT, as was demonstrated by the literature and our previous studies [7,11,12,13,14,15,16]. As an alternative to structural changes, the use of pharmaceutical nanotechnologies could be an optimal approach to ensure a good release of the PS molecule in the tumor tissues. 

Polymeric nanocarriers have the ability to enhance the solubility of the porphyrinic photosensitizer, offer greater capacity for hydrophobic dissolution, enhance bioavailability, and ensure controlled release in the tumoral tissues. Hydrophilic molecules like polyethylene glycol and polysaccharides are frequently used to enhance the solubility of porphyrin derivatives in biological media [17,18]. 

Polysaccharide-based biopolymer hydrogels, such as those derived from hydroxypropyl cellulose (HPC), offer a promising solution for photosensitizer delivery. These hydrogels are biocompatible and biodegradable, and can provide a moist environment conducive to wound healing [19]. Incorporating porphyrins into these hydrogels could enhance their stability and bioavailability, offering a controlled release system for topical PDT. 

Hydrogels, particularly those derived from biopolymers, have emerged as versatile delivery systems for various biomedical applications. Polysaccharide-based biopolymer hydrogels, in particular, are of great interest due to their biocompatibility, biodegradability, and ability to maintain a moist environment, which is beneficial for wound healing and tissue regeneration [20]. By incorporating porphyrin photosensitizers into these hydrogels, it is possible to create a controlled release system that enhances the stability and bioavailability of the photosensitizers while providing localized delivery for topical PDT.

Zeitouni et al. [21] provide information regarding the practical guidance for the utilization of the newest 5-aminolevulinic acid (ALA) product (ALA 10% gel) in PDT for actinic keratosis (AK) and selected non-melanoma skin cancers (NMSC). Topical PDT using aminolevulinic acid (ALA) or its methyl ester proved to be a frequent option for actinic keratosis and non-melanoma skin cancer treatment, but the clinical potential of ALA is limited by the low rate of its uptake in cells and its poor bioavailability, both attributed to its structural characteristics and hydrophilic nature [22]. 

**Figure 1:**
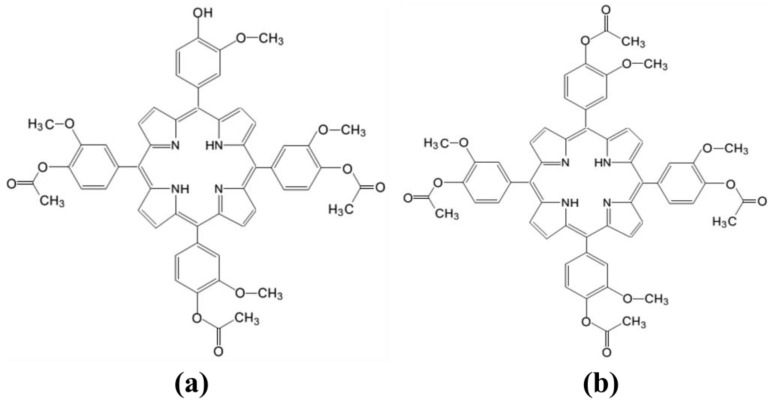
The molecular structure of the porphyrins under study: (**a**) 5-(4-hydroxy-3-methoxyphenyl)-10,15,20-tris-(4-acetoxy-3-methoxyphenyl) porphyrin (P2.2.), (**b**) 5,10,15,20-tetrakis-(4-acetoxy-3-methoxyphenyl) porphyrin (P2.1.).

This study investigates the incorporation of two porphyrin structures, 5-(4-hydroxy-3-methoxyphenyl)-10,15,20-tris-(4-acetoxy-3-methoxyphenyl) porphyrin (P2.2.) and 5,10,15,20-tetrakis-(4-acetoxy-3-methoxyphenyl) porphyrin (P2.1.) (Figure 1), with appreciable potential of cellular internalization and photochemical properties, into hydroxypropyl cellulose hydrogels. These photosensitizer/biopolymer systems were evaluated as potential therapeutic agents for topical photodynamic therapy. The hydrogels underwent comprehensive physicochemical characterization using Fourier-transform infrared (FTIR) spectroscopy, X-ray diffraction (XRD), thermogravimetric analysis (TGA), atomic force microscopy (AFM), UV-visible (UV-Vis) spectroscopy, and fluorescence spectroscopy. Additionally, pharmacotechnical evaluations were performed to assess the mechanical properties, pH, swelling ratio, and spreadability of the gels, aiming to establish their suitability for biomedical applications in PDT. 

In conclusion, the development objectives of our research are: (i) to obtain semi-solid pharmaceutical forms with PS of the tetrapyrrolic type, with a superior structural and spectral profile; and (ii) the pharmacotechnical and physicochemical evaluation of porphyrinic gels, with the aim of exploiting the therapeutic potential of the two porphyrins.

## 2. Results and Discussion

### 2.1. Physicochemical Characterization of the Gels

#### 2.1.1. FTIR Spectroscopy

**Figure 2:**
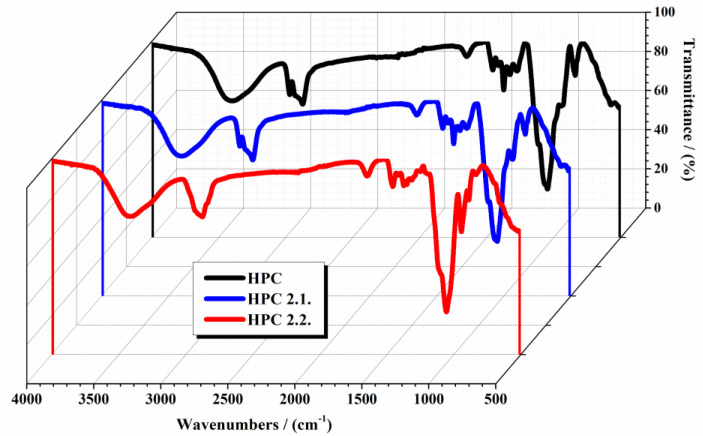
FTIR spectra of analyzed samples in the range of 4000–500 cm^−1^.

Figure 2 shows the FTIR analysis of the studied gels: 10% HPC gel, HPC P2.1., and HPC P2.2.

The FTIR spectrum of HPC from Figure 2 (black line) displays several characteristic adsorption bands according to the literature data: at 3400 cm^−1^ corresponding to the OH group from the pyranose unit, at 2970.8 cm^−1^ and 2874 cm^−1^ attributed to CH_2_ and CH stretching vibrations, at 1651 cm^−1^ due to C=C stretching vibrations, at 1374 cm^−1^ assigned to C-H stretching and deformation vibrations, and at 1048 cm^−1^ associated with C-O-C stretching vibrations [23,24,25]. Upon the incorporation of the P2.1. and P2.2. porphyrin into the polymeric hydrogel with 10% HPC, some changes in the original bands of HPC are observed, reflecting the interactions between the P2.1. and P2.2. porphyrin molecules and the HPC matrix (blue line and red line from Figure 2). A slight shift and a small change in the intensity of the peak from 3400 cm^−1^ due to the interaction between the OH groups of HPC and the two porphyrins (3401.82 cm^−1^ for HPC P2.1. and 3393.14 cm^−1^ for HPC P2.2.) was observed. The bands due to CH_2_ and CH stretching also show small changes due to the presence of additional methoxy and acetoxy groups from HPC P2.1. and HPC P2.2. The FTIR spectrum of HPC P2.2. shows the presence of a new small peak at 2900 cm^−1^. Also, the C-O-C stretching suffers a small alteration, reflecting new interactions between the HPC and porphyrin molecules. These observations confirm the successful incorporation of P2.1. and P2.2. into the HPC matrix and suggest significant interactions that might affect the gel’s structural properties. These modifications suggest potential rearrangements within the polymer network and new interactions, particularly hydrogen bonding and possibly other intermolecular forces, between the HPC and porphyrin molecules. Comparative analysis between the HPC P2.2. and HPC P2.1. gels indicate distinct interaction patterns. The observed spectral differences underscore the influence of different substituents on the porphyrin ring on the overall gel structure and properties. P2.2., with its additional acetoxy groups, shows more pronounced changes in the FTIR spectrum, suggesting stronger or more extensive interactions with the HPC matrix compared to P2.1. The incorporation of porphyrins into the HPC matrix and the resultant structural changes are crucial for understanding the physicochemical properties of these gels. The successful integration of porphyrins is evidenced by the shifts in existing bands.

#### 2.1.2. XRD Analysis

**Figure 3:**
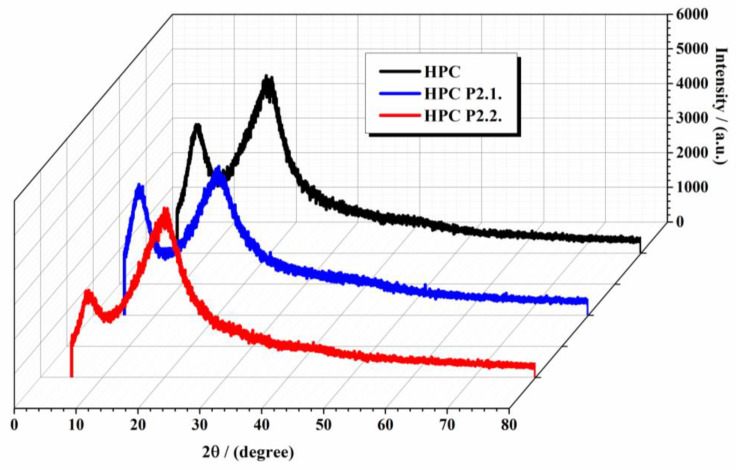
XRD diffractograms of analyzed samples.

The X-ray diffractograms of the two incorporated porphyrins in the HPC matrix compared with the 10% HPC gel are displayed in Figure 3. X-ray diffraction analysis provides crucial information on the crystalline structure and phase composition of studied gels. By comparing the XRD patterns of 10% hydroxypropyl cellulose gel with those of HPC gels incorporating P2.2. and P2.1. porphyrin, we can gain insights into the changes in structure and potential interactions between the polymer matrix and porphyrin molecules.

The XRD pattern of 10% HPC gel (Figure 3—black line) exhibits two broad diffraction peaks around 2θ° = 8 and 21°, characteristic of its semicrystalline nature. These broad peaks indicate a relatively low degree of crystallinity, with the majority of the polymer existing in an amorphous state. The lack of sharp peaks confirms the amorphous nature of the polymer, which is expected for HPC due to its hydroxypropyl groups disrupting regular packing [24,26,27]. The incorporation of P2.1. and P2.2. in the HPC matrix shows slight shifts and changes in intensities (the first peak from 2θ° = 7° shows a more pronounced decrease in intensity for HPC P2.2. compared with HPC P2.1., and the peak from 2θ° = 20° shows a more pronounced increase in intensity for HPC P2.2. compared with HPC P2.1.), indicating interactions between the porphyrins and the HPC matrix. The XRD analysis reveals the incorporation of two porphyrins into the 10% HPC gel matrix. The increase in the intensity of the peak from 2θ° = 20° in the HPC P2.2. sample can be evaluated as a partial crystallization of the porphyrins within the gel matrix. This can influence the material’s properties, including its mechanical strength, stability, and drug release behavior. The alteration of amorphous regions may enhance the controlled release of porphyrins, which is crucial for their application in topical photodynamic therapy.

#### 2.1.3. Thermal Analysis

**Figure 4:**
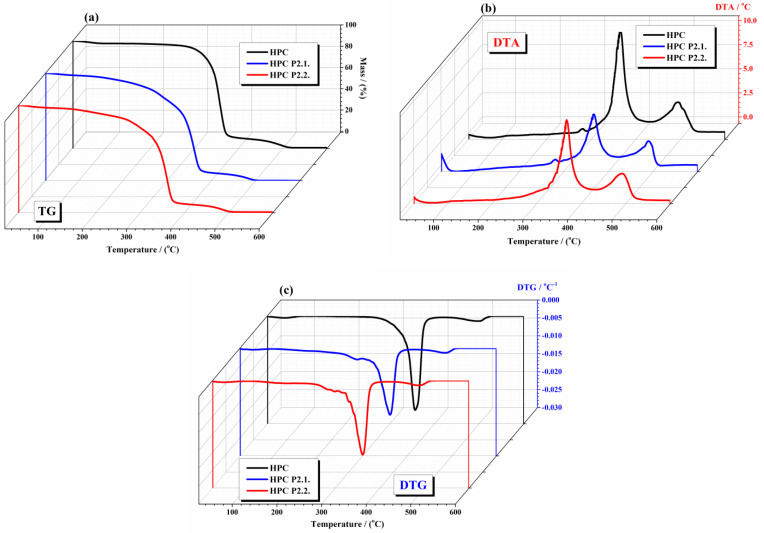
Thermal curves (TG, DTG, DTA) of studied gels: (**a**) TG curves (**b**) DTA curves; (**c**) DTG curves (experimental conditions: atmosphere—air at a flow rate of 0 mL min^−1^; heating rate—10 °C/min).

Thermogravimetric analysis (TGA) provides crucial insights into the thermal stability and decomposition behavior of these types of materials. By comparing the thermal profiles of 10% hydroxypropyl cellulose with those of HPC gels incorporating P2.2. and P2.1., it can understand how the presence of these porphyrins affects the thermal properties of the polymeric hydrogel systems. Figure 4 shows the thermal curves (TG, DTG, and DTA) of the studied gels: Figure 4a for TG analysis, Figure 4b for DTA analysis, and Figure 4c DTG analysis, obtained in the air atmosphere.

The TG curve of 10% HPC gel exhibits two main stages of weight loss: (i) in the first stage (below 100 °C), the initial weight loss is attributed to the evaporation of absorbed moisture and is minor (2.4%), indicating the loss of water physically bound to the polymer; (ii) the second stage between 250 and 550 °C shows two decomposition steps and a total weight loss in this range, which corresponds to the thermal degradation of the HPC polymer backbone. The decomposition involves the breakdown of glycosidic linkages and subsequent volatilization of low molecular weight products [28]. The thermal curves of HPC P2.1. and HPC P 2.2 displays a modified thermal profile compared to 10% HPC gel. Similar to HPC, for both HPC P2.1. and HPC P 2.2, the first stage corresponds to moisture loss. The interactions between HPC and P2.1 and P2.2. affected the moisture retention capacity of the gel. It was observed, in this stage, a weight loss of 1.8% for HPC P2.1. and 2.2% for HPC P2.2., respectively. The weight loss stage between 270 and 500 °C is presented in all samples, but shows variations in peak positions and intensities in the DTG and DTA curves, reflecting different degradation behaviors due to the porphyrins. It was observed that the presence of porphyrins slightly reduces the moisture content due to possible interactions between the polymer and porphyrin. The weight loss from the second range is influenced by both the HPC matrix and the decomposition of the P2.1 porphyrin. The DTG and DTA curves show additional peaks (at a peak temperature of 287 °C for DTG curves and 281 °C for DTA curves), indicating different degradation pathways or interactions between the polymer and porphyrin. The thermal data obtained from TGA experiments are represented in Table 1.

The incorporation of porphyrins enhances the thermal properties of the hydrogel HPC matrix, leading to more stable composite materials [29]. Enhanced thermal stability ensures that the gels maintain their structural integrity and functional properties under varying temperature conditions, which is crucial for storage, handling, and applications. The presence of porphyrins not only provides therapeutic benefits, but also contributes to the robustness of the hydrogel systems.

#### 2.1.4. AFM Results

**Figure 5:**
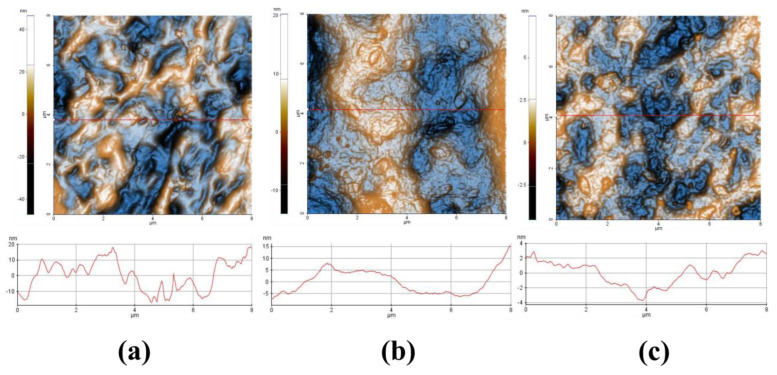
Two-dimensional AFM images, topography, at the scale of 8 µm × 8 μm of the 10% HPC (**a**), HPC P2.1. (**b**), and HPC P2.2. (**c**), accompanied by representative line scans, plotted at the position indicated in the AFM images by red horizontal lines.

Figure 5 shows the 2D topographic AFM images of the 10% HPC (Figure 5a), HPC P2.1. (Figure 5b), and HPC P2.2. (Figure 5c) samples, scanned over an area of 8 µm × 8 μm. In Figure 5a, the morphology of the 10% HPC hydrogel is evidenced, consisting of a random alternation of peaks (ridges) and valleys, leading to height differences in a few tens of nm (app 40 nm), as suggested by the line scan plotted in Figure 5a (the position is indicated by the horizontal red line in the AFM image). The 10% HPC sample is characterized by a Root Mean Square (RMS) roughness of 11.9 nm and a peak-to-valley height difference of 93.7 nm. The incorporation of the P2.1. porphyrin into the polymeric hydrogel with 10% HPC decreases the corrugation of the surface, as can be visually observed in the AFM image (Figure 5b), and the corresponding line scan (in which the vertical scale is of approx. 20 nm). The overall scanned area has an RMS roughness of ~4.6 nm and a peak-to-valley parameter of 34.3 nm. The incorporation of the P2.2. porphyrin into the polymeric hydrogel (Figure 5c) leads to a further decrease in the corrugation parameters so that the corresponding line scan presents a vertical scale of 7 nm (from −4 to +3 nm). The RMS roughness of the whole AFM image from Figure 5c was 1.3 nm, and the peak-to-valley parameter was 12.0 nm. 

**Figure 6:**
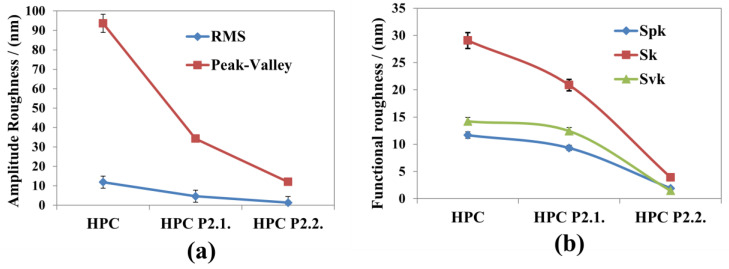
Roughness evaluation, 10% HPC, HPC-P2.1, and HPC-P2.2 samples based on Amplitude (RMS roughness and peak-to-valley) (**a**) and Functional parameters (Reduced Summit Height (Spk), Core Roughness Depth (Sk), and Reduced Valley Depth (Svk)) (**b**).

Figure 6 presents the evaluation of different roughness values, evaluated from amplitude parameters (Root Mean Square roughness and Peak-to-Valley) and functional parameters (Reduced Summit Height (Spk), Core Roughness Depth (Sk), and Reduced Valley Depth (Svk)) for the images presented in Figure 5. It can be observed that all roughness parameters decrease in the sequence: HPC > HPC P2.1. > HPC P2.2.

Core roughness depth (Sk) is the difference between the upper and lower levels of the core, the reduced summit height (Spk) represents the mean height of the protruding peaks above the core, while the Svk parameter (reduced valley depth) is the mean height of the protruding features beneath the core. A high Sk value here correlates with the geometrical corrugation of the surface (larger Rq and RMS roughness values). Spk is related to a surface composed of high peaks that provide large areas of contact (stress) when the surface is contacted (HPC sample). In other words, the Spk parameter can represent the height of material that can be removed during friction. Svk is a measure of the valley depth below the core roughness, and can be related to possible liquid retention (or debris pickup). The lowest values in the series (sample HPC P2.2.) may express promising mechanical properties (pharmacotechnical applications).

#### 2.1.5. UV-Vis and Fluorescence Spectroscopy

**Figure 7:**
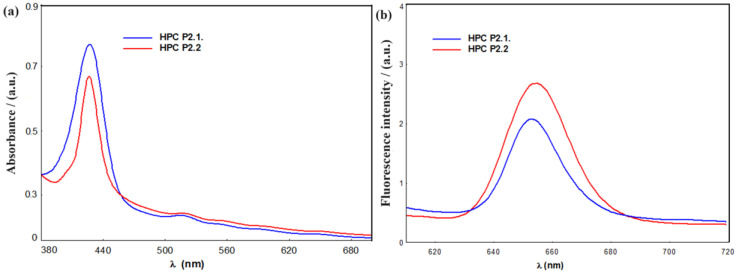
(**a**) UV-Vis spectra of the samples of, respectively, P2.1. (blue) and P2.2. (red) in HPC presented without normalization. (**b**) Fluorescence intensity vs. wavelength for the samples of, respectively, P2.1. (blue) and P2.2. (red) in HPC displayed without normalization.

Figure 7 reveals the UV-Vis (Figure 7a) and fluorescence (Figure 7b) spectra of the studied HPC P2.1. and HPC P2.2. samples.

The electronic absorption spectra of the samples HPC P2.1 and HPC P2.2 proved to be a typical porphyrinic profile [12]. Despite the fact that the samples are not obviously colored, from a spectral point of view, both porphyrins keep the strong coloration due to their highly conjugated 18-electron system π. The π-π* is still responsible for these types of spectra. Practically, the energy profile consists of a strong transition to the second excited state (S0 → S2) at about 426 nm (the *Soret* or B band), and a set of four weaker but still considerably visible signals, weak transitions to the first excited states (S0 → S1) in the region after 500 nm (the Q bands) [30,31]. Between these two categories of bands, the molar extinction coefficients are of one order of magnitude difference. Also, the presence of all four distinct Q bands proves the existence of the porphyrin in the originally synthesized form, with *D2h* symmetry as a free base. Moreover, both B bands appear to be very similar in position and intensity: at 426.71 nm for HPC P2.1. and at 426.10 nm for the HPC P2.2., as seen in Figure 7a. The rest of the spectrum has the expected profile with a mild decrease in intensity Q bands: 517.5, 550.9, 588.9, and 647.3 nm for the HPC P2.1., and 516.8, 556.3, 596.6, and 649.3 nm for HPC P2.2., respectively. In this particular case, the similar porphyrin produced, as expected, similar spectra with reduced differences. The absorption profile confirms the stability of the porphyrins in this frame of pharmaceutical formulae, with all of the derived characteristics remaining unaltered.

Regarding the fluorescence, the presence of this main signal (which in all cases is the strongest) is a result of the emission S1 → S0, which is the most exploited in this porphyrinic frame. It is centered, as expected, between 600 and 700 nm. The band at higher energy, corresponding to the S2 → S0 transition, usually does not appear due to multiple situations such as internal conversion or reduced quantum relaxation/return rate. The excitation at almost the same wavelength triggered similar responses in the cases of both gel samples. Practically, this intense signal of fluorescence is enough to establish the potential as a fluorescent marker; the 653.2 nm for HPC P2.1. and 654.2 nm for HPC P2.2. proved to be in a narrow interval of the wavelength, as expected for two structures with differences in the level of *meso*-substitutions. The intensity is strong, proving that the matrix has a weak to insignificant influence on P2.1. and P2.2. fluorescence.

The main target of these absorption and emission spectra was to establish (i) if the compound was present in this formula, (ii) if the matrix affected the structure of the porphyrin, which must be stable, (iii) to see if fluorescence properties are affected, and (iv) to obtain a comparison profile for other pharmaceutical formulae as related to HPMC, for instance. The conclusions are obvious for all the points: the porphyrin structure is completely unaffected by the components of this environment, and the pharmaceutical formulation is suitable for this point of view by the capacity to allow for porphyrin to display the whole fluorescence potential, which is one of the strongest points in medical use as a marker, for instance. Finally, it should be noted that the photophysical characteristics of these gel samples are linked to the structure of the porphyrins, and in the spectroscopic window used for these investigations, both UV-Vis and fluorescence, all other components are irrelevant.

### 2.2. Pharmacotechnical Evaluation of the Porphyrin Gels

#### 2.2.1. Wet Gels Evaluation

The obtained hydrogels were slightly pink, transparent, homogenous, and sticky, and no air bubbles could be observed. The density of 10% HPC gel was 1.15 g/mL; meanwhile, HPC P2.1. had a density of 1.14 g/mL, and HPC P2.2. had a more diminished density of 1.09 g/mL.

##### pH Values

The pH of the base gel was 5.5, but the addition of the porphyrins increased the value to 6.8 for HPC P2.1. and 6.7 for HPC P2.2. The two porphyrins induce a significant increase in pH, with similar values between the two formulations, but bring the final pH of the gels close to neutral. The pH values of the two formulations ensure good tolerability, with no irritation effects when applied to the skin [32].

##### Spreadability

**Figure 8:**
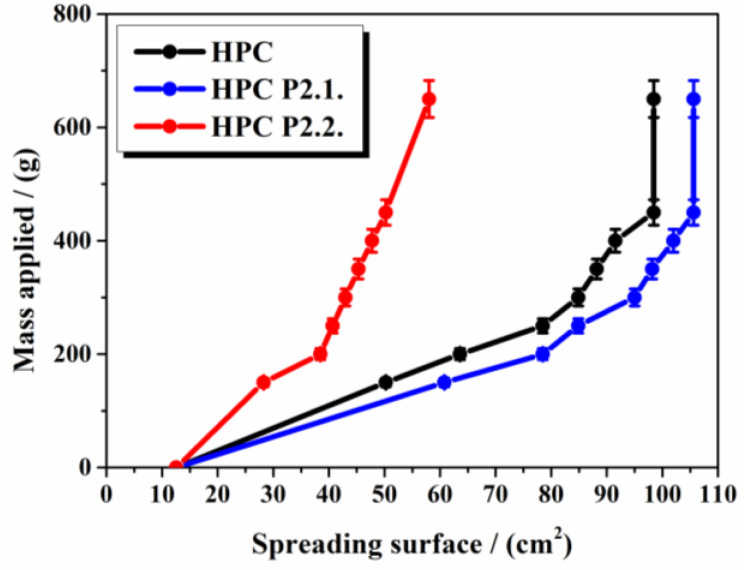
The spreading behavior of the hydrogels.

The variation of the spreading surface with the applied mass is shown in Figure 8.

It is noticed that porphyrins highly influence the spreadability of the HPC gel. While P2.1. slightly increased the spreading area, P2.2. considerably diminished the extension area of the gel. For HPC and HPC P2.1. hydrogels, a similar behavior is remarked, with a significant increase in the extensibility after the first 300 g were applied, then the spreading area extends less importantly up to 450 g applied, remaining unchanged for weight above 450 g. Meantime, HPC P2.2. gel shows a linear increase in the spreading area as a function of the applied mass and seems to continually extend when higher weights are used. The results prove that HPC P2.1. will probably quickly and evenly spread over the treated area, while HPC P2.2. will require additional shear stress to uniformly extend on the skin.

##### In Vitro Adhesion Ability

The results of the adhesion analysis are consistent with the findings from the spreadability assessment, with P2.2. possessing higher adhesion properties as the tensile force needed was 44 g/cm^2^. The required tensile force for HPC gel was 36 g/cm^2^ and for HPC P2.1. 32 g/cm^2^. Both porphyrins, even in small amounts, change the mechanical properties of the HPC base gel, but their influence is obviously different. While P2.1. has less influence in terms of reducing matrix cohesion resulting in better spreadability but lower adhesion, P2.2. significantly increases the molecular forces in the gel, resulting in diminished spreadability and a higher adhesion.

##### Rheology Measurements

**Figure 9:**
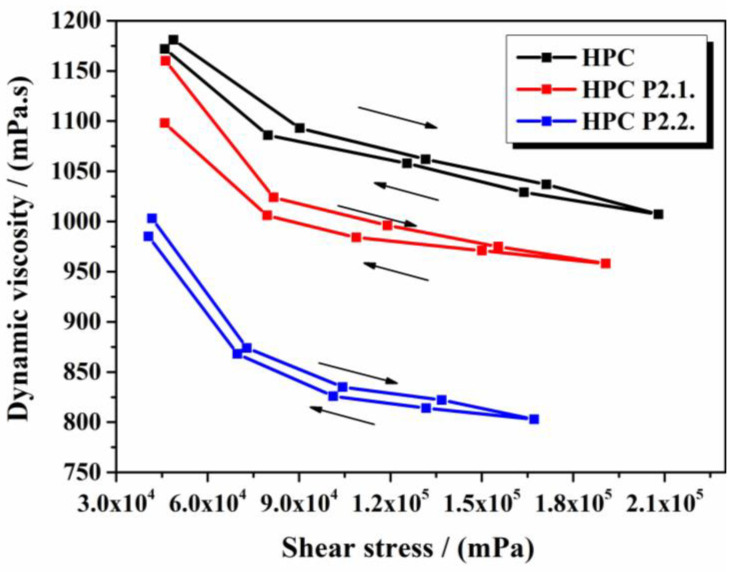
Dynamic viscosity vs. shear stress representation for the studied gels.

The rheological behavior of the studied gels is shown in Figure 9. 

The rheological analysis of the three investigated gels revealed important insights into their structural and flow properties, which are critical for their application in drug delivery and topical therapies. For the three gels investigated, a pseudoplastic behavior typical of gelled systems was observed, in which the shear stress increases and the dynamic viscosity decreases with increasing speed. Pseudoplasticity is advantageous for topical applications because it allows for the gels to become less viscous under shear (e.g., during application), facilitating easier spreading, and then recovering their viscosity once the shear is removed, maintaining their position on the skin. All samples show a thixotropic character with similar shear stress and dynamic viscosity in both the upward and downward shear rate directions. Thixotropy is beneficial in formulations intended for topical use because it indicates that the gel can recover its structure after shear deformation, ensuring consistent application and retention on the skin. The incorporation of P2.1. into the HPC gel structure does not lead to a significant change in the flow properties of the systems. For HPC gel and HPC P2.1 gel, the values of shear stress and dynamic viscosity are comparable, which proves that P2.1 does not interfere with the matrix arrangement of the gel. The structural integrity and flow properties remain intact, which is desirable for maintaining the gel’s performance while delivering the active compound. In the case of P2.2., the behavior is different, since both the shear stress and the viscosity decrease compared to the base gel, revealing a clear change in the system structure and consequently in the viscoelastic manifestation. The incorporation of P2.2. into the polymer matrix has led to modifications in the internal structure of the material, to spatial distribution rearrangements, or new interactions [33,34,35]. 

##### Dry Gels Evaluation

The thickness of the films is about 0.06 ± 0.003 mm, with no obvious differences between the porphyrin gels and the HPC base gel. The uniformity of the film is necessary, since the thickness correlates directly with the concentration of the active ingredient and the bioadhesion function of the polymer [36,37]. Furthermore, the tensile strength of the films varied between 0.61 kg/mm^2^ for HPC P2.1, and 0.57 kg/mm^2^ for HPC P2.2. and 0.49 kg/mm^2^ for the HPC base gel, suggesting that the porphyrin load increases the resistance of the film. This can be explained by the plasticizing effect of PEG 200 [38], the solvent used for the solubilization of the two porphyrins, which increases the viscoelastic properties of the gel matrix and thus its mechanical properties. In addition, the elongation percentage is enhanced from 13% for HPC gel to 18% for HPC P2.1 and 15% for HPC P2.2., indicating good flexibility of the pharmaceutical products, which is probably provided by the plasticizer PEG 200. The moisture content of HPC P2.1 was 8.21%, similar to the HPC base gel with 8.03% humidity. Meanwhile, the moisture content for HPC P2.2. was higher, being 10.35%. These findings explain the higher adhesive properties and lower viscosity of HPC P2.2. The linked water forms hydrogen bonds with the accessible chains of the HPC, enabling them to move more freely [39] and reducing the viscosity of the system.

**Figure 10:**
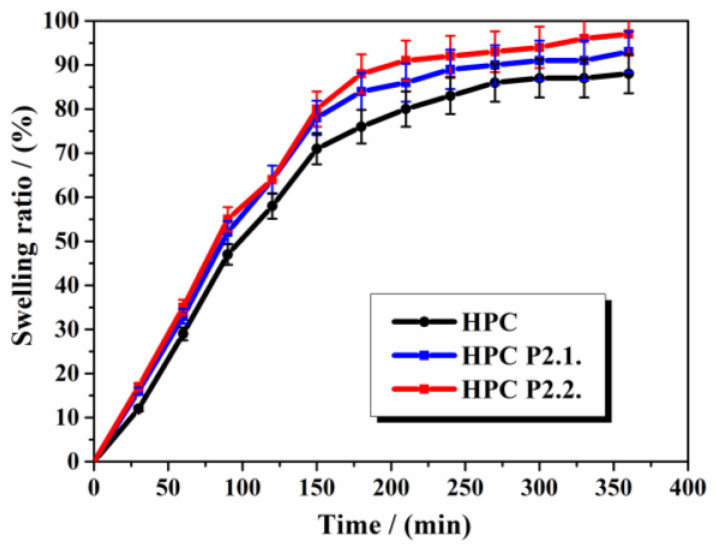
The swelling rate of the studied gels.

The swelling rate over 6 h is shown in Figure 10.

The three series of hydrogels showed similar swelling behavior, with a significant absorption of humidity in the first 150 min, then the ratio slowly increased up to 88% for HPC gel, 93% for HPC P2.1. gel, and 97% for HPC P2.2. after 360 min. The effect of porphyrins on the swelling behavior of the HPC matrix is not meaningful, proving that the ability to absorb moisture is determined by the polymer.

### 2.3. Discussion

According to Rizwan M et al. [40], the pH value of hydrogels influences their physico-chemical properties as well as their release and biological performance. Nevertheless, as a non-ionic polymer, HPC retains its gelling properties in a wide pH range between 2 and 11. However, its stability is affected by highly acidic or alkaline media, which can degrade over time. HPC is known for its good biocompatibility with human skin, and is even suitable for application to damaged skin [41]. Even if the two porphyrins raise the pH value considerably, the stability and tolerability characteristics for HPC are maintained.

Spreadability is an important mechanical property of gels that reflects the ease and uniformity of application on the skin. It is one of the most significant qualities in topical application, as it can influence the therapeutic efficacy and patient adherence to treatment [42]. Extensibility performance is the result of the product cohesiveness and P2.1. was found to maintain the structural characteristics of the HPC matrix, offering only a slight increase in spreading ability. On the contrary, P2.1. changed the alignment of the polymer molecules, creating stronger cohesive forces in the network and significantly reducing the flexibility of the system. 

It was found that the cohesiveness of the hydrogels is significantly influenced by the type of porphyrin dispersed in the polymer matrix. It can be observed that P2.1. does not interfere so much with the gel structure, but P2.2. clearly generates new strong cohesive forces in the system. Ferrari et al. [43] demonstrated that the gel strength is associated with the degree of crosslinking in the polymer matrix. The results lead to the idea that HPC P2.2. will induce a longer contact time with the skin, but HPC P2.1. will be applied much more easily on the tegument surface. 

Rezvanian et al. [44] proved that ionic crosslinking enhances the mechanical strength of the gels, but their study demonstrated that lower levels of crosslinking lead to better drug release properties. Also, Radulescu DM et al. [45] explained that the mechanical properties of the gel depend on both the stiffness of the polymer chains and the density of crosslinking through intermolecular interactions. Chen X et al. [46] showed that to enhance the adhesive properties of a hydrogel, either more hydrogen bonds are formed or other crosslinking methods are involved. The adhesion qualities of polymer gels are significantly influenced by their viscoelastic nature [47]. 

There is a high correlation between the spreading, adhesive, and viscoelastic properties of the HPC P2.1. gel, similar to the HPC base gel. In contrast, an inconsistent relationship between the parameters is achieved for HPC P2.2. While the spreadability is lower and the adhesion is stronger than for the other two systems, it would be expected that the viscosity would be higher, but the measurements showed it to be lower. HPC P2.2. behavior can only be explained by the influence of P2.2. on the matrix structure by creating new interparticle bonds that considerably change the rheological attributes. Some other studies mentioned similar results for different gel types [48,49], and most of the authors considered it a filling effect of the active ingredient on the polymer matrix. For HPC P2.2., this may not be the case, as its concentration is extremely low. As other authors stated [50,51,52], dehydration could be the cause of this discrepancy. 

Ghebremedhin M et al. [53] explained the increased deformation by the transfer of more deformation energy, which occurs before some portions of the internal structure are distorted and the connections between the particles are broken. Long network bridges that are not anchored in the network permanently may also be the cause of these findings and observations [54,55]. Since it may interact with the suspended chains of the gel particles, the water that is exuded from the gel surface may affect the rheological properties. In this way, the free chains are able to hold this released water through hydrogen bonds. The dynamic of the released water is quick, and the speed and mobility of the water molecules determine how fast they diffuse to the surface [56]. 

The analyses on the dry films give a better insight into the behavior of the hydrogels when they are applied to the skin. The prepared hydrogels will form a thin and uniform film on the skin surface, which proves that the porphyrins are suitable for incorporation into the HPC matrix. According to Nair AB et al. [36], the ideal thickness range for bioadhesive films is 0.05 to 1.0 mm. Pünnel LC and Lunter DJ [57] pointed out that the film formation is highly dependent on the selected polymer, as its chains interact between them when the solvent evaporates, creating a solid polymer matrix that is responsible for uniform adhesion and release of the active ingredient on the skin surface [58]. The viscosity of the hydrogel also plays an important role in the film’s thickness, as it increases during the solvent evaporation [59]. 

Hou C et al. [60] demonstrated that the interpenetrating polymer chains considerably enhance the elongation at break. A flexible and elastic film structure was also favored by the amorphous character and the high-water content. This is consistent with the findings of Nur Hazirah MASP et al. [61], who proved that the amorphous nature of the gel improved the elongation of the film when a plasticizer was added. The role of the plasticizer is to change the viscoelastic attributes of the polymer, which in turn affects the adhesion and behavior of the material upon release. 

The type and quantity of plasticizers and polymers used determine the resistance of the film, which is reflected in the tensile strength values. In water-soluble polymers, the crosslinking density improves with decreasing chain flexibility, preventing overhydration [62]. Functional groups capable of hydrogen bonding enhance the bioadhesion mechanism. A soft and weak polymer has low tensile strength and elongation values, a hard and brittle polymer typically has medium tensile strength and low elongation values, and a soft and tough polymer has high tensile strength and elongation values, according to Bharkatiya et al. [63].

The mechanical properties of the films are critical factors for topical hydrogels, as they indicate how resistant the material is to shape changes without developing cracks. Plasticizers have the ability to interact with the functional groups of the polymer by interposing themselves between its chains. As the intermolecular forces between the polymer chains are diminished, the bonds are weaker and the film’s flexibility improves as a result [64,65]. Repka et al. [66] declared that HPC has lower water affinity in comparison to other gel-forming polymers. Still, by adding P2.2. in the hydrogel matrix, the amount of retained water is increased, probably due to the modification in the gel structure [26,67,68,69,70]. 

The results for the swelling performance of HPC gels are in line with the findings of Cremer G. et al. [26], who also proved that the hydration rate and the surface hydrophobicity are strongly correlated; a higher surface hydrophobicity were linked to a quicker hydration rate. Although the swelling ratios are similar between the studied hydrogels, it can be noticed that the incorporation of P2.2. into the system leads to a higher and faster swelling rate, indicating the formation of new links in the gel structure.

Essentially, the substituted groups in the polymer establish how the active ingredients influence the swelling properties of the polymer matrix. The integrity of the matrix of swelling hydrophilic cellulose polymers depends on the hydroxyl group of the molecules [71]. The rate at which the active ingredient is released is largely determined by the swelling index. The swelling capacity of the polymer matrix is based on how well it resists the movement of water molecules. Water penetration is difficult for a polymer chain with a limited hydrogen bonding capacity, as it cannot form a robust matrix structure. This was demonstrated by Panomsuk et al. [72]. As the number of hydrogen bonds and the strength of the polymers increase, the diffusion of water molecules decreases. In addition, the swelling behavior of the films influences their bioadhesive efficacy.

Our findings demonstrate that the porphyrin-doped HPC hydrogels maintained desirable physicochemical properties such as high swelling ratios, appropriate mechanical strength, and consistent rheological behavior. These properties are crucial for topical applications as they ensure the hydrogel remains stable, provides adequate moisture to the wound site, and can be easily applied and retained on the skin. In terms of solubility and dispersion, the incorporation of porphyrins into the HPC matrix showed promising results. These results suggest that the hydrogel matrix acts as a protective and solubilizing environment for the porphyrin molecules. By embedding the porphyrins within the hydrogel, their tendency to aggregate is reduced, thereby enhancing their solubility in biological media. This improved dispersion, and stability could lead to better uptake by tumor cells, increasing the efficacy of PDT.

The FTIR spectra confirmed the successful incorporation of porphyrins into the HPC matrix, with characteristic absorption bands indicating interactions between the porphyrins and the polymer. These interactions suggest potential hydrogen bonding and integration of porphyrins within the gel structure. XRD analysis revealed that the porphyrins were effectively integrated into the HPC hydrogels, contributing to their stability and functional properties. Additionally, TG/DTG analyses demonstrated the thermal stability of the hydrogels, ensuring their stability under physiological conditions. TGA results showed that the thermal stability of the HPC–porphyrin gels was enhanced compared to 10% HPC gel. The presence of additional weight loss stages corresponding to porphyrin degradation indicates that the porphyrins contribute to the overall stability and robustness of the hydrogels. AFM imaging provided detailed topographical information, showing a homogeneous distribution of porphyrins within the hydrogel network, which is crucial for consistent therapeutic effects. UV-Vis and fluorescence spectroscopy confirmed that the porphyrins retained their photophysical properties post-incorporation, essential for their effectiveness in PDT. Rheological assessments demonstrated pseudoplastic behavior and thixotropic properties typical of gelled systems.

This comprehensive evaluation confirms that these porphyrin-incorporated HPC hydrogels have the potential to be effective therapeutic agents for topical PDT in the treatment of various disorders. Future research should focus on in vivo evaluations to validate the efficacy and safety of these hydrogels in order to establish the effectiveness and safety of these hydrogels in human subjects. Continued optimization of the hydrogel formulation may enhance its therapeutic properties and patient compliance. Additionally, investigating other polysaccharide-based biopolymers could provide insights into alternative delivery systems for porphyrins and other photosensitizers. In conclusion, this study presents a promising advancement in the field of topical PDT, providing a foundation for future research and potential biomedical applications in the treatment of various skin conditions and superficial tumors.

## 3. Conclusions

This study successfully demonstrates the incorporation of two porphyrin structures, 5-(4-hydroxy-3-methoxyphenyl)-10,15,20-tris-(4-acetoxy-3-methoxyphenyl) porphyrin (P2.2) and 5,10,15,20-tetrakis-(4-acetoxy-3-methoxyphenyl) porphyrin (P2.1), into hydroxypropyl cellulose hydrogels. Comprehensive physicochemical and pharmacotechnical evaluations were performed to assess the suitability of these hydrogels for use in topical PDT. The incorporation of P2.1. had minimal impact on the flow properties of the HPC gel, while P2.2. significantly altered the viscosity and shear stress, indicating structural modifications within the gel matrix. The hydrogels exhibited suitable mechanical properties, including appropriate rigidity and elasticity, essential for practical application on the skin. The pH of the hydrogels was found to be within the optimal range for topical applications, minimizing the risk of irritation. High swelling ratios were observed, indicating good water absorption capabilities, which are beneficial for maintaining a moist wound environment. Excellent spreadability was noted, ensuring easy application and even coverage on the skin surface.

In summary, our study indicates that porphyrin-doped HPC hydrogels not only retain desirable physicochemical properties, but also address key limitations of porphyrin derivatives in PDT. These findings support the potential of these hydrogels as effective carriers for porphyrins in topical photodynamic therapy, warranting further investigation into their biological performance and therapeutic efficacy.

## 4. Materials and Methods

### 4.1. Materials 

The porphyrins under investigation were acquired using the procedures that were previously detailed by our team [12]. Polyethylene Glycol 200 (PEG 200) and hydroxypropyl cellulose (HPC) were purchased from Sigma-Aldrich Chemie GmbH, Taufkirchen, Germany. All ingredients were weighed on a Mettler Toledo AT261 balance (with 0.01 mg sensitivity).

### 4.2. The Formulation and Manufacturing Process of the Hydrogels

HPC was chosen as the gel-forming polymer due to its advantages such as its water solubility, non-ionic nature, physiological inertness, non-irritancy, bioadhesiveness, and biodegradable qualities [73,74,75]. A 10% HPC hydrogel was obtained by dispersing HPC into water and mixing it at 800 rpm at room temperature using a Heidolph MR 3001 K magnetic stirrer (Schwabach, Germany). For de-aerating, the hydrogel was kept overnight in the fridge at 5 °C. The obtained gel served as a reference in the subsequent analyses and also as a base for the studied porphyrins’ gels. In total, 1 g of each 10 mM porphyrin solution in PEG 200 was separately added to 50 g of base gel and stirred in the dark at 750 rpm and at room temperature. Finally, two hydrogels (HPC P2.1. and HPC P2.2.) containing 10 µg porphyrin/g were obtained. The properties of the obtained gels were evaluated in comparison to the simple 10% HPC hydrogel.

### 4.3. Physicochemical Characterization of the Gels

Fourier-transform infrared (FTIR) spectroscopy was performed using a JASCO FTIR 4100 spectrophotometer (Tokyo, Japan). The spectra were recorded over the range of 4000–400 cm^−1^.

X-ray diffraction (XRD) analysis was conducted using a Rigaku Ultima IV diffractometer (Rigaku Co., Tokyo, Japan). The instrument operated in parallel beam geometry with CuKα radiation (λ = 1.5406 Å). XRD diffractograms were analyzed in the 2θ range of 5° to 60° at a scanning speed of 2°/min and a step size of 0.02°.

Thermogravimetric analyses were performed using a Mettler Toledo TGA/SDTA851^e^ thermogravimeter (Mettler-Toledo, Greifensee, Switzerland) under synthetic air flow at 80 mL/min with a heating rate of 10 °C/min. 

Atomic force microscopy (AFM) measurements were performed in “non-contact” mode with XE-100 (Park Systems, Suwon, Republic of Korea) equipped with decoupled sample/probe scanners, thus having a weak tip–sample interaction. All AFM measurements were performed with NSC36B tips (MicroMasch, Neuchatel, Switzerland), having a typical radius of curvature of less than 8 nm, full cone angle of ~40°, height ~15 µm, thickness of ~1 µm, length of ~90 µm, width of ~32 µm, force constant of ~2 N/m, and resonance frequency of ~130 kHz. The AFM figures are presented in the so-called “increased contrast” mode. In the “increased contrast” color scheme, the color of a pixel is determined by the color variation of neighboring pixels. For AFM investigations, a drop from each hydrogel-based sample was placed on clean microscope glass substrates (Heinz Herrenz Medizinalbedarf GmbH, Hamburg, Germany) and let to dry under room conditions. The images were processed with the XEI program (v 1.8.0, Park Systems) for display purposes (tilt correction). Several roughness parameters were evaluated: from amplitude parameters, root-mean square roughness (Rq—the standard deviation of the height value in the image) and the peak-to-valley parameter (Rpv—the height difference between the lowest and the highest points in the surface); and, respectively, from the functional parameters, as reduced summit height (Spk—the height of the material in the peak zone), core roughness depth (Sk—the height difference between the intersection points of the found least mean square line), and the reduced valley depth (Svk—the height of the valley zone). 

For UV-Vis recordings, the LAMBDA 35 UV/Vis Spectrophotometer by *Perkin Elmer Life and Analytical Science*^®^ (Perkin-Elmer, Waltham, MA, USA) —source Deuterium and Tungsten pre-aligned lamp with automatic switch-over, with high accuracy (Wavelength At D2 peak (656.1 nm ± 0.1 nm) and reproducibility (Wavelength 10 measurements at 656.1 nm ± 0.05 nm)—checked before experiments were used. The baseline flatness was also checked at the used 1 nm open slit, which was ±0.002. UV-Vis absorption spectra were measured using the single holder device of the spectrophotometer, optics single-beam sealed quartz-coated mirrors with a lens-free system to reduce chromatic aberrations. 

The very high accuracy of measurements is also due to the Solid Construction CNC-machined aluminum chassis for thermal and vibration stability and the extensive use of Circulator Bath–Thermoregulator FALC^®^ to ensure the thermal water circulation at the constant normal human body temperature at 37 °C.

All optical investigations were carried out in 1 cm quartz cuvettes using the samples as received; the same high-precision quartz fluorescence cell was used to carry the emission spectra. No dilution or other interventions were unfolded. 

The JASCO^®^ FP-6500 (JASCO Co., Ltd., Kyoto, Japan) used to perform the fluorescence experiments is a research-grade spectrofluorimeter with a minimum 1 nm resolution and a highest sensitivity S/N ratio of 200 or greater. Built-in low-pressure Mercury lamp and UV cut filter enable the GLP/GMP compliance with validation software available as standard. The light source is the lamp with a shielded lamp house, 150 W. 

The photometric ratio system uses a monochromatic light to monitor the intensity output of the Xe source (using a photomultiplier tube as the monitoring detector of the light incident). The high performance is defined by the sensitivity 3200:1 signal-to-noise ratio of the Raman band of water at 350 nm excitation wavelength, 2 s. The response time and 5 nm bandwidth for both excitation and emission monochromators was greater than 200:1 (peak-to-peak). The resolution was 1 nm on both Excitation and Emission. The excitation wavelength was set at 426 nm, according to the previous UV-Vis recorded spectra.

For the temperature control in the single cell holder, the Advantage—Lab AL03-10^®^ thermal circulation was used to maintain, during calibrations and measurements, the constant normal human body temperature at 37 °C. 

### 4.4. Pharmacotechnical Evaluation of the Porphyrin Gels

#### 4.4.1. Wet Gels Evaluation

##### pH Determination

In total, 0.2 g of each hydrogel was diluted with 1 mL of distilled water (pH 6.5 ± 0.5), then the pH values were recorded by using the electrode of a CONSORT P601 pH-meter (manufactured by CONSORT^nv^, Turnhout, Belgium).

##### Spreadability

In total, 1 g of each hydrogel was placed on a 2 cm diameter circle in the center of a glass plate. A second glass plate weighing 150 g weight was placed on the surface, and the diameter of the circle occupied by the gel was recorded. Weights of 50 g, 100 g, 150 g, 200 g, 250 g, 300 g, and 500 g were then successively placed on the upper glass plate, and the diameter of the circle occupied by the gel was determined after a resting time of 2 min. The spreading area engaged by the hydrogel was calculated using the following formula: S = πr^2^. 

##### In Vitro Adhesion Ability

A 1 cm^2^ area of one clamp of a Lloyd Instruments Ltd. digital tensile force tester (Lloyd Instruments Ltd., LR 10K Plus, West Sussex, UK) was covered with a thin layer of hydrogel, then the second clamp was applied to squeeze the gel. The test was performed at a speed of 100 mm/minute, and the mass required to release the second brace from the gel surface was recorded. The tensile force required to separate the second slide from the entire surface occupied by the hydrogel is a measure of the adhesion behavior and was calculated [76] using this Equation below:

(1) $$Tensile\;force\left(g/{mm}^{2}\right)=\frac{Detaching\;mass\left(g\right)}{Surface\left({mm}^{2}\right)}$$

##### Rheology Measurements

The experiments were performed on 50 g of each gel, using the RV5 measuring spindle of a B-one Plus rotational viscometer from Lamy Rheology Instruments, Champagne au Mont d’Or, France. The measurements were carried out at 22 °C at increasing rotation speeds, from 50 rpm to 250 rpm, then again in decreasing order. The duration of each determination was set at 150 s, and there was no break between two consecutive determinations. The dynamic viscosity was recorded and the hysteresis curve was plotted.

##### Dry Gels Evaluation

To determine a more objective performance of the gels after their application on the skin, the hydrogels were poured in a thin layer into Petri dishes and left to dry at a room temperature of 22 °C for 24 h. The dry films were then peeled off, and their mechanical and swelling properties were examined.

##### Mechanical Properties

Thickness

A digital micrometer from Yato Trading Co., Ltd., Shanghai, China, with a measuring range of 0 to 25 mm and a resolution of 0.001 mm, was used to determine the thickness of the formed film.

##### Tensile Strength and Elongation

The tensile strength and elongation behavior were assessed using a digital tensile force tester for universal materials made by Lloyd Instruments Ltd., LR 10K Plus, West Sussex, UK. The test was performed at a 20 mm distance and a speed of 3 mm/s. By placing the films vertically between the two braces, the breaking force could be measured [62]. The following formulas were used to determine the tensile strength and elongation at break:

(2) $$Tensile\;strength\left(kg/{mm}^{2}\right)=\frac{Force\;at\;breakage\left(kg\right)}{Film\;thickness\left(mm\right)\;\times \;Film\;width\left(mm\right)}$$

(3) $$Elongation\%=\frac{Increase\;in\;film\;length}{Inital\;film\;length}\times 1$$

##### Moisture Content

A Mettler-Toledo GmbH, Greifensee, Switzerland, HR 73 Mettler Toledo halogen humidity analyzer was used to assess the loss on drying according to the thermogravimetric method [24].

##### Swelling Ratio

In total, 0.2 g of film from each series were incubated at 37 ± 1 °C in Petri dishes containing 1.5% agar gel. For six hours, the films were weighed every thirty minutes. The following Equation was used to calculate the swelling ratio:

(4) $$Swelling\;ratio=\frac{Wt-Wi}{Wi}\times 100$$

where W_t_ is the patch weight at time t after the incubation and W_i_ is the initial weight [77,78,79].

## Figures and Tables

**Table 1:** The thermal data obtained from TGA curves.

Compound	1st Step (Temperature and Mass Loss)	2nd Step (Temperature and Mass Loss)	Remaining Mass at 600 °C
HPC	Below 100 °C/2.4%	*T*_DTA_ = 365 °C and *T*_DTG_ = 355 °C *T*_DTA_ = 495 °C and *T*_DTG_ = 501 °C	No residue
HPC P2.1.	Below 100 °C/1.8%	*T*_DTA_ = 367 °C and *T*_DTG_ = 357 °C *T*_DTA_ = 491 °C and *T*_DTG_ = 487 °C	No residue
HPC P2.2.	Below 100 °C/2.2%	*T*_DTA_ = 367 °C and *T*_DTG_ = 361 °C *T*_DTA_ = 492 °C and *T*_DTG_ = 489 °C	No residue

## Data Availability

Data are contained within the article.
